# A Systematic Review of Neuroprotective Strategies during Hypovolemia and Hemorrhagic Shock

**DOI:** 10.3390/ijms18112247

**Published:** 2017-10-26

**Authors:** Marius Nistor, Wilhelm Behringer, Martin Schmidt, René Schiffner

**Affiliations:** 1 Department of Neurology, Jena University Hospital, 07747 Jena, Germany; rene.schiffner@med.uni-jena.de; 2 Emergency Department, Jena University Hospital, 07747 Jena, Germany; wilhelm.behringer@med.uni-jena.de; 3 Institute for Biochemistry II, Jena University Hospital, 07747 Jena, Germany; Martin.Schmidt@med.uni-jena.de; 4 Orthopedic Department, Jena University Hospital, 07747 Jena, Germany

**Keywords:** neuroprotective strategies, neuroprotection, brain damage, hypovolemia, shock, haemorrhage, bleeding, resuscitation

## Abstract

Severe trauma constitutes a major cause of death and disability, especially in younger patients. The cerebral autoregulatory capacity only protects the brain to a certain extent in states of hypovolemia; thereafter, neurological deficits and apoptosis occurs. We therefore set out to investigate neuroprotective strategies during haemorrhagic shock. This review was performed in accordance to the PRISMA (Preferred Reporting Items for Systematic Reviews and Meta-Analyses) guidelines. Before the start of the search, a review protocol was entered into the PROSPERO database. A systematic literature search of Pubmed, Web of Science and CENTRAL was performed in August 2017. Results were screened and evaluated by two researchers based on a previously prepared inclusion protocol. Risk of bias was determined by use of SYRCLE’s risk of bias tool. The retrieved results were qualitatively analysed. Of 9093 results, 119 were assessed in full-text form, 16 of them ultimately adhered to the inclusion criteria and were qualitatively analyzed. We identified three subsets of results: (1) hypothermia; (2) fluid therapy and/or vasopressors; and (3) other neuroprotective strategies (piracetam, NHE1-inhibition, aprotinin, human mesenchymal stem cells, remote ischemic preconditioning and sevoflurane). Overall, risk of bias according to SYRCLE’s tool was medium; generally, animal experimental models require more rigorous adherence to the reporting of bias-free study design (randomization, etc.). While the individual study results are promising, the retrieved neuroprotective strategies have to be evaluated within the current scientific context—by doing so, it becomes clear that specific promising neuroprotective strategies during states of haemorrhagic shock remain sparse. This important topic therefore requires more in-depth research.

## 1. Introduction

Severe trauma is the leading cause of death and disability for patients below 45 years of age [1], and costs the lives of five million people per year [2]. While the specific mechanisms underlying the acquisition of these traumas differ significantly, both civilian and military injuries and accidents inherently include the risk of extensive bleeding. Exsanguination constitutes the most common cause of death in patients who die at the scene of the accident, and haemorrhage is a contributing factor in 33% to 56% of pre-hospital deaths after trauma [1]. Patients that present with severe haemorrhagic shock without a traumatic genesis are often suffering from gastrointestinal bleeding or placenta abruption [3,4].

Even survivors of haemorrhagic shock often face prolonged courses of recovery or long-lasting secondary health problems, as is evidenced by the fact that traumatic injuries globally constituted 11% of disability-adjusted life years in 2010 [5]. Severe haemorrhagic shock leads to a multitude of macro- and microcirculatory changes, oxygen free radical generation and subsequent cell death [3]; furthermore, even after resuscitation, damages can occur due to ischemia-reperfusion injury [6] and multi organ dysfunction syndrome [7]. While the cerebral autoregulation protects the oxygen and energy supply of neuronal cells during physiologic blood pressure fluctuations [8], severe hypovolemia caused by haemorrhagic shock exceeds the cerebral autoregulatory capacity [9], which causes malperfusion and neuronal damages [10]. Further investigations suggest that cerebral cortical structures are more susceptible to sustain damages during the aforementioned states as compared to the subcortex [11,12,13]—whether this is due to an inherent higher vulnerability on the cellular level as compared to the subcortex, or whether subcortical structures are better protected at the lower limits of the cerebral autoregulation, is still unclear. Since the cerebral cortex is essentially the centre of human cognition and thereby responsible for the enactment of adequate social responses to exterior influences [14], cortical damages often negatively influence the patients’ ability to return to work and maintain or return to the previously lived lifestyle. Apart from the devastating personal consequences, this poses a socio-economic stress on society as well. This is especially relevant in today’s highly specialised work environment, and might transform into an even bigger stressor, should the job market become more competitive and demanding due to a narrowing of existing labour opportunities [15].

To our knowledge, potential neuroprotective strategies during haemorrhagic shock and states of hypovolemia have not yet been the subject of a systematic review. We therefore decided to conduct a systematic literature search of three medical databases to retrieve studies relevant to this topic. Since “neuroprotection” is a very broad area of interest, we initially did not limit our interests exclusively to either human randomised controlled trials or experimental studies, expecting the eventual size of the results to be rather low. Since we were not able to retrieve any randomised controlled studies of human participants that adhered to our inclusion criteria, the following review exclusively describes neuroprotective strategies investigated in animal experimental studies.

## 2. Results

### 2.1. Study Selection

**Figure 1:**
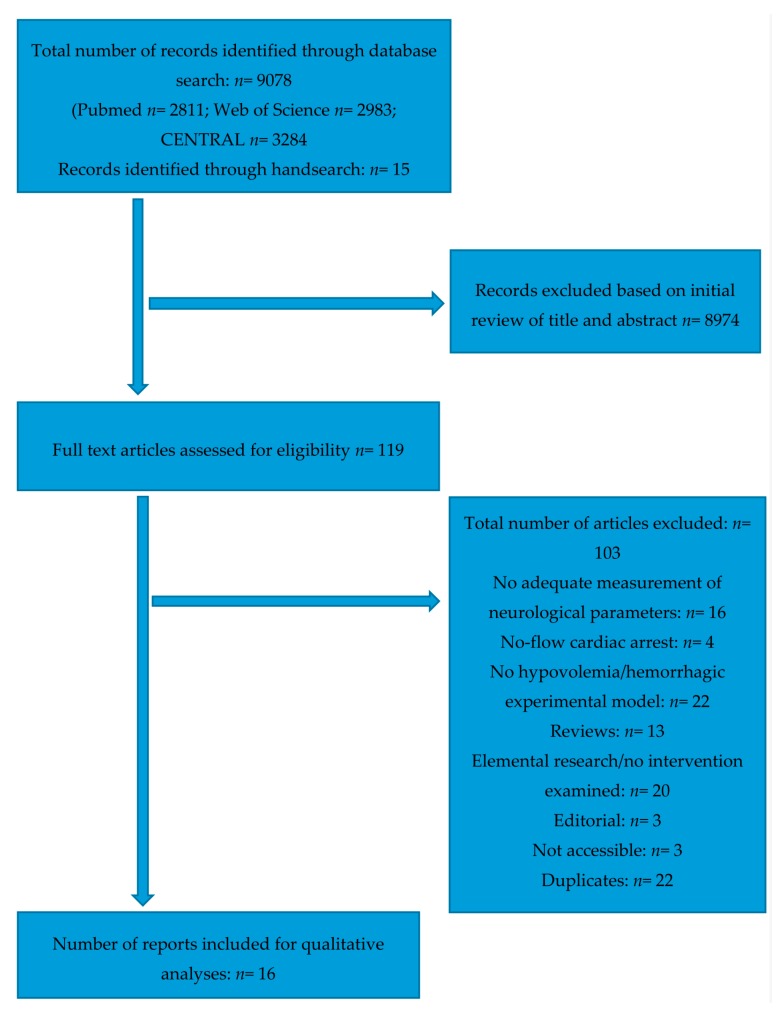
PRISMA (Preferred Reporting Items for Systematic Reviews and Meta-Analyses) flow chart.

The systematic literature search of the three databases yielded 9078 results in total, while 15 studies were identified through hand-search. Of these preliminary results, 8974 were excluded based on screening of title and abstract. The remaining 119 studies were retrieved for full-text assessment. After evaluation based on our inclusion criteria as described in the “Methods”, 16 studies remained for qualitative assessment. The complete search process is illustrated in the PRISMA (Preferred Reporting Items for Systematic Reviews and Meta-Analyses) flow chart (Figure 1).

### 2.2. Study Characteristics

All studies were written in English, published at the time of evaluation and performed in a laboratory setting.

Only animal experimental studies could be retrieved through the systematic literature search. Nine studies used pigs [16,17,18,19,20,21,22,23,24], five studies used rats [25,26,27,28,29], one study used dogs [30], and one study used cats [31]. The average number of animals used across the studies was 29.4 ± 12.4 (mean ± SD), the lowest number of utilised animals was 14 [22], and the highest was 60 [23].

All studies employed a hypovolemia model, albeit the extent of the defined hypovolemia criteria varied within individual studies. Eleven studies defined the hypovolemia by the meeting of predefined vital parameters, for example a specific MAP (mean arterial pressure) or HR (heart rate) [16,17,18,19,20,21,22,25,29,30,31], while the rest of the studies removed a certain amount of blood, either based on millilitres per kilogram, or on the estimated total blood volume [23,24,26,27,28].

The interventions assessed within the studies can be divided into three subsets: (1) hypothermia [23,26,31]; (2) (hypertonic) fluid solutions with the application of either epinephrine or vasopressin [16,17,18,19,20,21,22]; and (3) other interventions—which only yielded one result per intervention per study—which contain studies evaluating the neuroprotective properties of aprotinin [25], remote ischemic postconditioning [28], sevoflurane [27], piracetam [30], human mesenchymal stem cells [29] and Na^+^/H^+^ exchanger (NHE1) inhibition [24].

Essential vital parameters such as blood pressure and heart rate were measured by all studies. The evaluation of the effectiveness of the neuroprotective intervention was based on varying parameters within the studies, which include brain specific parameters [16,17,18,19,20,21,22,23,24,31], brain specific histo-pathological assessment [23,25,27,29,30], assessment of cognitive function [24,27,28,29], as well as other brain specific tissue parameters [19,20,21,22,23,25,26,27].

An overview of the individual study characteristics is provided in Table 1.

### 2.3. Results of Individual Studies

#### 2.3.1. Hypothermia

Guven et al. examined the effects of mild (32 °C) and moderate (28 °C) hypothermia, as compared to normothermia, during hypovolemia on reduced glutathione (GSH) levels and thiobarbituric acid-reactive substances (TBARS) in the brain stem tissue of rats. Moderate hypothermia was shown to maintain constant levels of GSH and to reduce the increase of TBARS, as compared to normothermia and mild hypothermia [26]. This led to the conclusion that moderate hypothermia exerts antioxidant protective effects, which could be indicative of a neuroprotective effect since oxygen radicals are known to cause a cascade of toxic oxidative reactions.

By Vogt et al., who investigated the neuroprotective influence of mild therapeutic hypothermia (33 °C) in a porcine model of multiple trauma with haemorrhagic shock, the temporal application of hypothermic efforts was shown to have a significant influence on the positive outcome of the intervention [23]. Employing a somewhat more realistic experimental model, the hypothermic intervention began either 90 or 120 min after haemorrhagic shock was established, was continued over 12 h and was followed by a 10 h rewarming period and a subsequent observation period of 48.5 h. Ultimately, positive effects in the form of reduced cerebral inflammation and less iNOS positive cells (as a marker of neurodestructive mircoglia polarisation) could only be observed in animals in which the hypothermia was started 90 min after established haemorrhagic shock, but not in animals which received cooling after 120 min [23].

In the context of these rather positive results, the study by Kishi et al. [31] provides a rather cautionary note, by assessing the effect of hypothermia on the cerebral autoregulation during hypovolemic hypotension in cats. Microscopic video recording through cranial window technique was utilised to investigate the vasodilatory response of pial arterioles during decreasing MAPs. Hypothermic animals with a temperature of 32 °C exhibited a significantly reduced vessel diameter of either large or small arterioles at MAP values of 50, 60, and 70 mmHg, as compared to the normothermic control group [31]. This absence of vessel dilation was interpreted as an impairment of the cerebral autoregulatory response through the hypothermic treatment.

Details of the different hypothermic interventions are summarised in Table 2.

Temporal information of hypothermic interventions are given as numeric values (in minutes = min/or hours = h) respective to the beginning of hypovolemia within the study design; negative values signify that hypothermia preceded hypovolemia, positive values signify that hypothermia was employed subsequent to hypovolemia.

#### 2.3.2. Varying Fluid Resuscitation Protocols and Hypertonic-Hyperoncotic Solutions, Partly in Combination with Epinephrine and Vasopressin

The majority of the results identified through the literature search in regard to fluid resuscitation protocols originate from what appears to be one particular research group, with two studies from Cavus et al. [16,17] and three from Meybohm et al. [20,21,22] (with a large overlap of the same researchers between all studies). All of the studies utilised the same porcine experimental model, which mimics the possible time line of an accident—beginning with the induction of haemorrhagic shock (=accident), followed by the intervention for 30 min (=pre-hospital initial fluid resuscitation), with a subsequent cessation of the haemorrhagic shock and observation period (=in-hospital surgical treatment). Investigating various combinations of hypertonic-hyperoncotic solution (HHS), norepinephrine (NE) arginine vasopressin (AVP) and fluid solutions, their main insights can be summed up by a superiority of HHS over normal fluid solutions. HHS in combination with AVP seemed to induce an additional benefit, as compared to HHS combined with NE. Both HHS + AVP and HHS + NE increased cerebral perfusion pressure (CPP) and middle arterial pressure (MAP) faster than fluid solutions after trauma, the greatest increase was observed in the HHS and AVP combination [17]. A similar temporal benefit of the increase of CPP and MAP was observed in a study comparing HHS combined with normal saline (NS) infusion with HHS + AVP and HHS + NE (HHS + AVP > HHS + NE > HHS + NS), albeit the initial advantages disappeared after 10 min [21]. Relatedly, HHS + AVP was initially superior in regard to CPP increase and cerebral oxygenation as compared to fluid solutions, but exhibited comparable cerebral metabolism rates and the same amount of secondary cell damage [20]. In a comparison between HHS + AVP and HHS + NE, the former was initially able to increase cerebral venous partial oxygen pressure, but again this initial superiority vanished after 10 min and ultimately both interventions exhibited comparable results in regard to brain metabolism [22]. Neither low-dose nor high-dose epinephrine in combination with HHS was found to exhibit superior results regarding CPP, brain tissue oxygen pressure and brain oxygen saturation, when compared with stand-alone HHS [16].

Evaluating the synthetic analogue of vasopressin, terlipressin, Ida et al. utilised a porcine model of haemorrhagic shock as well. Comparing terlipressin administration with Ringer solution application, it was shown that the former was able to normalise CPP and apoptosis, oxidative damage and cerebral markers of water damage [19].

Chien et al. compared a range of infusion protocols in a piglet model. All groups received an initial bolus of normal saline (NS) after haemorrhagic shock, while the three treatment groups received subsequent pressure-dependent infusions of either whole blood (WB), lactated ringer’s solution (LR) or NS. All groups receiving subsequent perfusions exhibited an improved cerebral tissue oxygenation as compared to a single bolus of NS, with NS + WB exhibiting the best results in maintaining cerebral tissue oxygenation [18].

Details of the different resuscitation protocols are provided in Table 3.

#### 2.3.3. Other Neuroprotective Strategies

##### NHE1-Inhibition

Wu et al. [24] tested the viability of inhibition of the ph-regulatory protein NHE1 (Na^+^/H^+^ exchanger) as a neuroprotective strategy and a means to maintain vital organ blood flow in pigs through NHE1-blockade with BIIB513 during haemorrhagic shock. NHE1-inhibition ameliorated vital organ blood flow and resulted in normal neurological outcome, most likely through an increased cardiac index with subsequently increased tissue perfusion [24].

##### Aprotinin

Eser et al. evaluated the effects of the serine protease inhibitor aprotinin in rats, and reported decreases of superoxide dismutase levels, malondialdehyde and myeloperoxidase levels, as well as apoptotic cells in aprotinin-treated animals as compared to a control group [25].

##### Piracetam

Piracetam addition to blood solutions, as performed by Özkan et al., in a haemorrhagic experimental model in dogs, only improved blood pressure and pulse rates as compared to blood solutions alone, albeit no significant differences were observed in either nitric oxide levels or obtained histopathological brain samples [30].

##### Sevoflurane

Hu et al. evaluated the neuroprotective effects of post-conditioning with sevoflurane in rats by utilising the Morris Water Maze test and expression levels of choline acetyltransferase (CHAT) and acetylcholinesterase (ACHE) as, respectively, measures of cognitive function and markers of the cholinergic system that have been identified as correlating factors to learning and memory capabilities [27]. Concentrations of 2.4% and 3.6% sevoflurane (but not 1.2%) were shown to significantly increase spatial learning and memory ability outcome three days after induced haemorrhagic shock, as well as to increase CHAT and to decrease ACHE expression [27].

##### Human Mesenchymal Stem Cells

Plaschke equally utilised a rat model of haemorrhagic hypotension with subsequent Morris Water Maze test to assess cognitive functions after human mesenchymal stem cell infusion [29]. A clear benefit on cognitive spatial learning could be observed as compared to control animals, although no marked structural immunohistological changes could be observed.

##### Remote Ischemic Preconditioning

Hu et al. investigated both the neuroprotective effects and the underlying mechanism of remote ischemic preconditioning (RIPC) in a rat model of haemorrhagic shock. Employing four 5-min cycles of limb ischemia, with and without K_ATP_ channel blockade, the neurological deficit score was significantly better in RIPC-treated animals after a 72 h observation period [28]. Since these beneficial effects were completely absent in K_ATP_ channel blocked animals, evidence for the underlying physiological mechanism of neuroprotection through RIPC was furthermore provided.

Details of the other neuroprotective strategies are listed in Table 4.

### 2.4. Risk of Bias within Studies

The risk of bias within studies was assessed with SYRCLE’s risk of bias tool [32], in the manner described in the “Methods” section.

A breakdown of the individual categories of SYRCLE’s risk of bias tool led to the following results: 100% of the included studies reported baseline characteristics; 37.5% reported allocation concealment; none of the studies provided information about random housing (albeit this category was only applicable to four studies); 25% reported performance blinding; none of the studies reported blinded outcome assessment; 62,5% reported detection blinding; 75% showed a low risk of incomplete data outcome; 75% showed a low risk of selective outcome reporting; 81.1% showed a low risk of bias in sequence generation; and 75% showed a low risk of bias in regard to other sources of bias.

The assessment outcome of the individual studies varied widely: the highest risk of bias was found in the studies of Eser et al. [25] and Plaschke et al. [29]; the majority of studies exhibited a medium risk of bias, while three studies—Cavus et al. [16], Meybohm [22] and Wu [24] —presented a very low risk of overall bias.

Of note is that, while 37.5% of the studies reported efforts of randomisation during assignment into experimental groups, only one study specifically described the randomisation procedure [19]. Similarly, only one of the four studies that mentioned efforts to performance blinding actually substantiated this with a description of the blinding efforts during enactment of the experimental protocol [24].

The results of all studies in regards to SYRCLE’s risk of bias tool are visualised in Table 5.

## 3. Discussion

Through a systematic literature search of three medical databases, we were able to identify a number of animal experimental studies investigating neuroprotective effects during states of haemorrhagic shock. No human randomised controlled trials that adhered to our inclusion criteria could be identified. Taking into account that our “intervention”—category was very loosely and broadly termed neuroprotection, the number of results we were ultimately able to retrieve appears to be quite low. Further research is required to investigate the consequences of haemorrhagic shock on cerebral structures and to find therapies preventing the concomitant cell damage.

### 3.1. Reviewed Interventions

The question which type of fluid solutions is more beneficial in critically ill patients has been a subject of controversial discussion for a number of years. While the studies of Cavus et al. and Meybohm et al., utilising hypertonic solutions, seem to suggest a positive correlation between both MAP values and cerebral parameters as opposed to normal saline [16,17,20,21,22], multiple meta-analyses and reviews on human subjects have not been able to replicate the benefits that hypertonic solutions have exhibited in animal models [33,34,35]. Current guidelines recommend isotonic crystalloid solutions for the treatment of hypotensive non-TBI patients [36]; not only has there been no positive evidence for a general administration of hypertonic solutions in trauma patients, under certain conditions 28-day mortality was even found to be increased [37].

While norepinephrine (NE) is recommended in current guidelines in combination with fluid therapy as a last resort to maintain the MAP during critical hypovolemic states [36], a routine application of vasopressors is nonetheless not recommended and some studies even suggest a deleterious influence if utilised instead of fluid administration [38,39]. If specific neuroprotective properties would be found after norepinephrine administration—and currently more insight is warranted for such a statement—these would still have to be evaluated critically against the general haemodynamic influence of the substance.

Arginine vasopressin and terlipressin were found to exhibit neuroprotective properties in the respective animal models of Ida et al., Cavus et al., und Meybohm et al. [17,19,20,21,22]. While vasopressin is currently not included in general guidelines for the treatment of haemorrhagic shock, a variety of animal studies and a number of provisional human studies have reported general positive outcomes after AVP administration, such as overall survival and effectiveness of vascular tone management [40,41]. In vasodilatory shock through sepsis, a review of nine randomised controlled trials even noted potential advantages over norepinephrine treatment [42]. AVP exhibits some advantages over norepinephrine that are especially relevant during haemorrhagic shock states, such as its better effectiveness in low-ph environments [40]. While AVP currently appears to be quite promising, further studies need to be performed to ensure its overall safety and to explore its differing physiologic responses. While our systematic review has yielded results in which AVP had a beneficial effect on cerebral parameters, Anand et al. notes contrasting results observed in other studies—AVP’s effects appear to be heavily dependent on dosage and physiologic characteristics at the time of administration, such as ph-imbalance, which furthermore emphasises the need for in-depth analysis of the substance [40].

Three of the retrieved studies investigated hypothermia as a means to specifically protect cerebral cells during hypovolemic states. Hypothermia as a neuroprotective intervention has been the focus of countless studies within the last decades, and has been applied to a variety of pathologic states, for example different neurological illnesses, post-resuscitation therapeutic hypothermia and traumatic brain injury [43,44]. Even though hypothermia initially generated enthusiasm due to very positive results in a multitude of animal experimental models, its translational value in human subjects has not been as positive, with many hypothermic interventions unable to reproduce the erstwhile success exhibited in laboratory settings [43]. While Guven and co-workers’ and Vogt and co-workers’ hypothermic interventions appear to be promising within the confines of their respective experimental models [23,26], the results should be seen in the greater history of ambiguous reproducibility of therapeutic hypothermia. Noteworthy is that Guven and co-workers’ study simultaneously began both execution of the hypovolemia and the hypothermia protocol—the study model therefore does not reflect a realistic temporal application of a hypothermic intervention, which Vogt et al. demonstrate to be a vital condition. Kishi et al. similarly induced hypothermia even 60 min before the start of the intervention, which equally somewhat questions the methodology and translational potential of the experimental model [35]. Apart from these abstract cautionary notes, there appear to be some strong contraindications to hypothermic interventions in the haemorrhagic patient. Hypothermia is known to negatively influence coagulation and haemostasis [45]. As a result, several guidelines for the treatment of polytrauma patients recommend normothermia and argue to avoid core temperatures below 34 °C [46,47]. Kishi and co-workers’ study points towards an additional physiologic response to hypothermia that might even negatively influence the cerebral blood flow during haemorrhagic shock [31]. Further considerations about hypothermic interventions have to include the possibility of an increased infection risk, although the prevalence of pneumonia after therapeutic hypothermia remains ambiguous [43].

Currently, the contraindications, whether clear (haemostasis and coagulation) or unclear (infection risk), seem to outweigh the contemporary state of evidence for neuroprotective benefits of hypothermia.

While both fluid solutions and hypothermia are intrinsically linked to the state of haemorrhagic shock—although hypothermia not necessarily as an obvious treatment solution but primarily as a pathogenic mechanism—Eser et al., Plaschke et al., Özkan et al., and Wu et al. represent independent approaches to neuroprotective strategies. While already considered for their respective neuroprotective properties, piracetam [48,49], NHE1-inhibition [50,51], human mesenchymal stem cells [52,53] and aprotinin [54,55] all appeared to exert neuroprotective properties within their respective haemorrhagic models [24,25,29,30]. Wu and co-workers’ observed normal neurological outcome of the intervention group as compared to the control animals might not only be traced back to an improved cerebral blood flow, though, since NHE1-inhibition can furthermore delay the cascade of energy depletion that results in tissue injury [28]. Even more unclear are the exact mechanisms that exert neuroprotective properties after human mesenchymal stem cell administration, although trophic factors might be a potential explanation [33]. While the results appear interesting, further research is warranted since these studies only represent the first studies to investigate these properties (as identified by our systematic literature search) in haemorrhagic shock models. Furthermore, three of the four studies exhibited high (2) or medium (1) risk of bias.

Neuroprotection through anaesthetic agents has been a focus of research for a number of years. Many animal experimental models show clear benefits utilising a variety of anaesthetic agents [56]—such as sevoflurane by Hu et al., as identified by us [27]. Schiffilliti et al. notes that neuroprotective effects are likely dose-dependent [56], which Hu and co-workers’ model seems to confirm [27]. Ishida et al. nonetheless cautions that exact mechanisms through which neuroprotective effects are mediated remain unanswered, and that the general quality of research concerning the neuroprotective properties of anaesthetic agents should be improved [57].

Hu and co-workers’ study, investigating both the neuroprotective properties and the physiologic mechanisms of remote ischemic preconditioning, constitutes the application of a relatively new and certainly interesting procedure in the context of a haemorrhagic shock model [28]. Remote ischemic pre/post-conditioning (RIPC) is defined as short cycles of induced extremity ischemia (of differing lengths in different protocols) with the objective to induce tolerance to reperfusion injury in another bodily region, for example the brain or the heart, through activation and modulation of humoral factors, inflammatory responses to reperfusion and mitochondrial permeability [44,58]. Various target illnesses and regions that could potentially benefit from RICP are currently under investigation [59,60,61]. Hu and co-workers’ study utilised a preconditioning protocol: although clinically, post-conditioning would be needed for states of traumatic haemorrhagic shock, pre-and post-conditioning appear to be similarly effective in a multitude of experimental models [58]. While certainly an enticing and promising intervention especially due to its simplicity, further research is required, in particular in the context of haemorrhagic shock. As Meller et al. notes, multiple general questions remain regarding RICP, for example in terms of the number of applications (equally in regard to a single application as to long-term use) and also in the choice of application site (leg vs. arm) [58]. Furthermore, whether even short cycles of limb ischemia could be deleterious in patients already suffering from haemorrhagic shock and centralisation remains to be investigated.

### 3.2. Study Quality and Translational Value

The quality of the included studies varied widely. Only four studies exhibited an overall low risk of bias, while the bulk of the remaining experiments ranged in the medium risk of bias category. This fact is rather sobering, when placed into the context of potential translational applications of the evaluated interventions. De Vries et al. emphasises the importance of systematic reviews of animal experimental studies as a precursor for the implementation of subsequent preclinical and clinical studies, but at the same time notes the importance of bias-free and methodologically correct experimental models as a requirement for the success of this objective [62]. As already noted in the methods section, while few studies did mention efforts of randomisation into experimental groups and performance at all, respectively only one group in each category substantiated this with a description of the randomisation/blinding procedure. Regarding the methodological approach, two characteristics of the experimental models many of the investigated studies exhibited should be noted: (1) the intervention was initiated immediately after hypovolemia was established/hypovolemia criteria were met; and (2) very few experimental models featured a prolonged observation period. We believe that these two aspects are noteworthy, because they do not adequately reflect the events of a clinical hypovolemia. Since most haemorrhagic events unfold in a preclinical setting, immediate treatment will not be accessible in the most cases. Similarly, cerebral alterations after a hypovolemic event unfold on a temporal level, which often comprises multiple days [7]. A very short observational period is thus prone to miss more gradual (but nonetheless critical) cerebral alterations.

The variety of utilised outcome measurements furthermore complicates comparability between different experimental models and likely multiplies the number of necessary experiments until a potential intervention can be considered for translational human studies. A consensus within the research community would be desirable, that agrees upon a set of minimally included neurologic parameters for the evaluation of neuroprotective interventions, which would have to be furthermore dependent on the utilised species.

Therefore, we believe that future studies should be more attentive in both describing and executing the procedures that prevent risk of bias, as well as utilising methodologically consistent experimental models that reflect the clinical situation more comprehensively. These efforts would not only enhance the potential translational benefits, but also serve for the additional implementation of the 3R principle (especially refinement and reduction) [63].

### 3.3. Limitations

Albeit we were careful to design our literature search to be quite comprehensive, we cannot entirely exclude the possibility of having missed results that were outside of our search protocol. Initially, we had planned to also perform a comprehensive literature search of the EMBASE database; unfortunately, we were unable to gain access to this database through the means available to us at our institution.

In order to identify as many potential neuroprotective interventions as possible, we did not include survival time and mortality in our inclusion criteria, since we anticipated that this would diminish the number of potentially eligible studies (which proved true, since few of our retrieved studies noted these observations). These aforementioned parameters will obviously have to be included in future, more comprehensive efforts to evaluate this subject, since overall survival represents a superior outcome measurement as a whole, as compared to neuroprotection.

Since our review was designed to provide an overview of the current research state of neuroprotective strategies during haemorrhagic shock, our results include a variety of differing interventions, which did not lend themselves for statistical analysis or meta-analysis. While we identified certain subsets within the totality of the results, we similarly decided against statistical efforts since we felt that such a depiction would inherently remain incomplete within the summary nature of the review. The individual qualitative descriptions of the interventions should therefore not be understood as recommendations or endorsements of treatment options, but as an abstract depiction of current ideas to approach the topic, especially since the initial concept of this review was not designed to produce a valuation.

## 4. Materials and Methods

### 4.1. Review Protocol

Before the execution of the literature search, a detailed review protocol was created in accordance to the “Preferred Reporting Items for Systematic Review and Meta-Analysis Protocols” [64]. As recommended by the PRISMA-P guidelines, the review protocol was registered with the PROSPERO database (Prospero-ID: 42017074770, August 2017).

### 4.2. Eligibility Criteria

As recommended by the PRISMA statement [65], we initially structured the systematic review with the help of the PICOS acronym (i.e., Participants, Interventions, Comparators, Outcome measures, Study design).

The PICOS criteria were identified as follows:

- *Types of participants*: Experimental animal models of haemorrhagic shock and hypovolemia, as well as studies considering human participants suffering from the same conditions.
- *Types of interventions*: All neuroprotective interventions.
- *Types of Comparators*: Trials comparing the interventions either with a control group/no intervention, standard care or other neuroprotective interventions (if the study design featured multiple interventions).
- *Types of outcome measures*: A neuroprotective effect, either measured through neurological parameters, cognitive tests or imaging techniques (brain imaging, staining, etc.).
- *Types of study design*: Randomised controlled trials.

All published studies were considered that were published between the years of 1995 and 2017 and were written in the English language.

We did not specify the exact extent of haemorrhagic shock or hypovolemia, since we did not want to significantly limit our inclusion criteria as we already anticipated the number of retrieved reports to be relatively low. Furthermore, it can be argued that any study that reports cerebral damages in the control group has an innately sufficient hypovolemia/haemorrhagic model. For the same reasons as stated before, we did not define any temporal requirements or limits for the haemorrhagic shock/hypovolemia.

Exclusion criteria were firstly defined as any significant aspects of a study that did not adhere to the PICOS characteristics we initially defined. Since we aspired to evaluate neuroprotective strategies specifically during states of hypovolemia and haemorrhagic shock, further exclusion criteria focused on any injury/illness models that would create an additional effect on the cerebral (patho)physiology. Any studies featuring animal models that, besides haemorrhagic shock/hypovolemia, included injury models such as traumatic brain injury, intracerebral haemorrhage, epilepsy, sepsis, spinal shock/injuries, or any other neurodegenerative diseases, were excluded. Similarly, human studies including traumatic brain injury, intracerebral haemorrhage, stroke, epilepsy, sepsis, spinal shock, or any other neurodegenerative diseases were excluded. Furthermore, in both animal models and in human studies, no-flow cardiac arrest was considered as an exclusion criterion.

### 4.3. Information Sources

Comprehensive literatures searches of Pubmed, Web of Science and CENTRAL were performed in August 2017. Filters employed in the database searches were language (English) and date of publication (1995–2017). Search terms revolved around variations of the following terms: “hemorrhagic shock”, “hypovolemia”, “neuroprotection”, “cerebral blood flow” and “randomised clinical trial”. In CENTRAL and Pubmed, MesH terms were employed in the literature search. The full search strategies for all three database searches can be accessed in the appendix (Appendix A).

Furthermore, a hand-search was performed in relevant journals and the reference lists of reviews focusing on the subject.

### 4.4. Study Selection

The complete lists of results of the three database searches were examined for eligibility in an unblinded manner by two reviewers. Disagreements were resolved through discussion and consensus.

### 4.5. Data Collection Process

We created a data extraction sheet that was based on the “Joanna Briggs Data Extraction Form for Experimental and Observational Studies” [66]. Data extraction was conducted in accordance to this previously defined extraction form in an unblinded manner, independently by two reviewers. Disagreements based on the extracted data were resolved through discussion until consensus was reached.

### 4.6. Data Items

The following data were extracted from the recovered trials:

1. General data of the respective trials (animal model, number of animals, and general vital parameters);
2. The specific hypovolemia model that the respective study used (i.e., blood loss/heart rate/blood pressure values the studies’ authors defined as an established hypovolemia);
3. The type of intervention (including dosage and duration);
4. The outcome measures of the neuroprotective interventions (brain specific parameters, cerebral histopathological values, cognitive tests, and further brain tissue markers).

### 4.7. Risk of Bias in Individual Studies

To evaluate the risk of bias in the retrieved studies, two reviewers independently assessed the risk of bias within the experimental procedure and the reporting of these studies, by means of answering the question’s proposed by SYRCLE’s risk of bias tool [32]. This tool for bias assessment is principally based on Cochrane’s risk of bias tool [67], but has been refined and adapted for use on experimental animal studies.

### 4.8. Summary Measures and Analysis

Due to our, intentionally wide-ranging, PICOS characteristics, the retrieved studies varied widely in regards to the specific interventions that they evaluated. Due to this variety of results, we decided against statistical efforts to compare the studies and against a meta-analysis. Although two subsets of the results (hypothermia, fluid therapy with vasopressors) consist of multiple studies, we decided against a statistical interpretation of these results as well, since our PICOS characteristics were not specifically modelled to retrieve results that specifically targeted these two interventions, and we suspected that such an interpretation might therefore render incomplete results. Further specific systematic reviews of these two topics might be warranted.

Our summary measures therefore take the form of a qualitative interpretation and a narrative analysis.

## 5. Conclusions

Our systematic literature search on neuroprotective therapies in haemorrhagic shock revealed only few animal studies. While the individual study results of hypertonic solutions, vasopressors, hypothermia and some neuroprotective substances showed a neuroprotective effect, human studies show conflicting evidence in terms of mortality. Specific promising neuroprotective strategies during states of haemorrhagic shock remain sparse. This important topic therefore requires more in-depth research. Further research is needed to investigate the consequences of haemorrhagic shock on neuronal structures and to find therapies preventing the subsequent damage.

## Figures and Tables

**Table 1:** **A/B.** General overview of included studies.

**Table 1A.** Study design.					
**Author/Year**	**Animal Model**	**Intervention**	**Total Number of Animals n = x**	**Extent of Hypovolemia**	**Observation Period of Animals after Intervention (None = Direct Euthanasia)**
Cavus et al., 2008 [16]	pigs	HHS vs. low-dose norepinephrine vs. high-dose epinephrine	22	MAP < 25 mmHg or −20% HR of baseline	None
Cavus et al., 2009 [17]	pigs	HHS vs. low-dose norepinephrine vs. high-dose epinephrine	24	MAP < 25 mmHg or −20% HR of baseline	None
Chien et al., 2011 [18]	pigs	normal saline vs. initial bolus saline and, respectively, whole blood, ringer lactat and normal saline	30	<45 mmHg MAP	None
Eser et al., 2007 [25]	rats	Isotonic solution vs. aprotinin	18	40–50% systolic BP of baseline	None
Guven et al., 2002 [26]	rats	Normothermia vs. 32 °C hypothermia vs. 28 °C hypothermia	40	40% of estimated total blood volume	None
Hu et al., 2014 [28]	rats	RICP vs. RICP and KATP-blocker	21	50% estimated total blood volume	to 3 days
Hu et al., 2016 [27]	rats	low-dose sevoflurane vs. moderate-dose sevoflurane vs. high-dose sevoflurane	35	Unclear	3 days
Ida et al., 2015 [19]	pigs	Ringer lactat vs. terlipressin	46	<40 mmHg MAP	None
Kishi et al., 2000 [31]	cats	Normothermia vs. hypothermia (in respect to vasodilatory response of pail arterioles	20	50 mmHg MAP	None
Meybohm et al., 2006 [20]	pigs	Fluid vs. HHS and arginine vasopressine	16	<25 mmHg or −20% HR of baseline	None
Meybohm et al., 2008 [21]	pigs	Crystalloid and colloidal fluids vs. HHS and NS vs. HHS and arginine vasopressine	30	<25 mmHg or −30% HR of baseline	None
Meybohm et al., 2007 [22]	pigs	HHS and norepinephrine vs. HHS and arginine vasopressin	14	<25 mmHg MAP or −20% HR of baseline	None
Özkan et al., 2008 [30]	dogs	Blood and saline vs., blood and piracetam	40	40 mmHg MAP	None
Plaschke 2009 [29]	rats	hMSC	32	30–40 mmHg MAP	6 days
Vogt et al., 2017 [23]	pigs	Hypothermia after 90 min vs. 120 min	60	40–50% of estimated total blood volume	None
Wu et al., 2012 [24]	pigs	NHE1 inhibtion	22	Removal of 40 mL/kg blood	3 days
**Table 1B.** Outcome parameters.					
**Author/Year**	**Detected Vital Parameters (e.g., Blood Gases, Hemodynamics, Blood Pressure, Heart Rate, Temperature)**	**Detected Brain Specific Parameters (e.g., ICP, CPP, CBF, Diameters of Brain Resistant Vessels, rScO2)**	**Detected Brain Specific Histo-Pathological Treatmens (Neuronal Cell Damage)**	**Detected Log Term Results, Cognitive Function**	**Other Specific Parameters in Brain Tissues**
Cavus et al., 2008 [16]	+	+	−	−	−
Cavus et al., 2009 [17]	+	+	−	−	−
Chien et al., 2011 [18]	+	+	−	−	−
Eser et al., 2007 [25]	+	−	+	−	SOD, MDA, MPO
Guven et al., 2002 [26]	+	−	−	−	GSH, TBARS
Hu et al., 2014 [28]	+	−	-	+	−
Hu et al., 2016 [27]	+	−	+	+	CHAT, ACHE
Ida et al., 2015 [19]	+	+	−	−	AQP4, NKCC1, SOD, TBARS, Bax, Bcl-x
Kishi et al., 2000 [31]	+	+	−	−	−
Meybohm et al., 2006 [20]	+	+	−	−	Glu, La, Py, Gly
Meybohm et al., 2008 [21]	+	+	−	−	S100B
Meybohm et al., 2007 [22]	+	+	−	−	Glu, La, Py, Gly
Özkan et al., 2008 [30]	+	−	+	−	−
Plaschke 2009 [29]	+	−	+	+	−
Vogt et al., 2017 [23]	+	+	+	−	S100B, NSE, Iba1
Wu et al., 2012 [24]	+	+	−	+	−

Abbreviations: cerebral blood flow (CBF); cerebral perfusion pressure (CPP); intracranial pressure (ICP); regional cerebral oxygen saturation (rScO_2_); glucose (Glu); lactate (La); pyruvate (Py); glycerol (Gly); remote ischemic preconditioning (RICP); Na^+^-H^+^ exchanger (NHE1); hypertonic-hyperoncotic hydroxyethyl starch solution (HHS); S100 calcium binding protein B (S100B); neuron specific enolase (NSE); inducible nitric oxide synthase (iNOS); adenosine triphosphate-sensitive potassium channel (K_ATP_); microglial reactivity marker (Iba1); human mesenchymal stem cells (hMSC); reactive oxygen species (ROS); superoxide dismutase (SOD); malondialdehyde (MDA); oxygen free radicals (OFRs); glutathione (GSH); thiobarbituric acid reactive substances (TBARS); aquaporin-4 (AQP4); Na^+^-K^+^-2Cl-co transporter (NKCC1); members of the apoptosis regulator protein Bcl-2 family (Bax and Bcl-x); myeloperoxidase (MPO); choline acetyltransferase (CHAT); acetylcholinesterase (ACHE).

**Table 2:** Specific characteristics of hypothermic interventions.

Author	Start of Hypothermia Respective to Hypovolemia	Depth of Hypovolemia	Length of Hypovolemia (Total Length/Time after which Desired Depth was Achieved)	Rewarming Period
Guven et al. [26]	0 min	32 °C vs. 28 °C	1.5 h/0.5 h	None
Kishi et al. [31]	−60 min	32 °C	1.4 h/?	None
Vogt et al. [23]	+90 vs. +120 min	33 °C	12 h/3 h	10 h

**Table 3:** Neuroprotective interventions with HHS or vasopressin or epinephrine.

Author/Year	Interventions	Dosage		Start of Intervention Respective to Hypovolemia	Length of Intervention/Length of Subsequent Observation Period
Cavus et al., 2009 [17]	Fluid resuscitation	6% HES 130/0.4, 20 mL/kg, and Ringer 40 mL/kg		Immediately after predefined hypovolemia criteria were met	30 min/30 min
	NE + HS	Bolus 20 µg/kg and continuously 1 µ/kg/min + HS 4 mL/kg			
	AVP + HS	Bolus 0.2 U/kg and continuously 0.04 U/kg/min + HS 4 mL/kg			
Cavus et al., 2008 [16]	HHS	4 mL/kg		Immediately after predefined hypovolemia criteria were met	30 min/30 min
	HHS + low-dose NE	4 mL/kg + 500 µg and 1/kg/min			
	HHS+ high-dose NE	4 mL + 1000 µg and 1 µg/kg/min			
Chien et al., 2011 [18]	NS	NS (all groups: 10 mL/kg	/	Immediately after induction of haemorrhagic shock	240 min/0 min
	NS + WB		15 mL/kg (+additional 15 mL/kg every 15 min if MAP > 45		
	NS + LR		15 mL/kg (+additional 15 mL/kg every 15 min if MAP > 45		
	NS + NS		15 mL/kg (+additional 15 mL/kg every 15 min if MAP > 45		
Ida et al.,2015 [19]	LR	Three-times of bled volume		30 min after predefined hypovolemia criteria were met	Instantenous/120 min
	Terlipressin	2 mg bolus			
Meybohm et al., 2006 [20]	Fluid resuscitation	Ringer’s solution (40 mL/kg) and hydroxyethyl starch 130/0.4 (20 mL/kg)		Immediately after predefined hypovolemia criteria were met	30 min/30 min
	HHS + AVP	4 mL/kg + bolus 10 U and continuously 2 U/kg/h			
Meybohm et al., 2008 [21]	Fluid resuscitation	Crystalloid (40 mL/kg) and colloid (20 mL/kg)		Immediately after predefined hypovolemia criteria were met	30 min/30 min
	HHS + NS	4 mL/kg + 10 mL bolus and continuously 1 mL/kg/h			
	HHS + AVP	4 mL/kg + 0.2 U/kg bolus and continuously 2 U/kg/h			
Meybohm et al., 2007 [22]	HHS + NE	4 mL/kg + 1000 µg bolus and continuously 60 µg/kg/h		Immediately after predefined hypovolemia criteria were met	30 min/30 min
	HHS + AVP	4 mL/kg + 10 U bolus and continuously 2 U/kg/h			

AVP, arginine vasopressin; HS, hypertonic starch solution; HHS, Hyperhaes; LR, lactated Ringer’s solution; NS, normal saline; NE, norepinephrine; WB, whole blood.

**Table 4:** Other neuroprotective strategies.

Author/Year	Intervention	Dosage	Start of Intervention Respective to Hypovolemia	Length of Intervention/Length of Subsequent Observation Period
Eser, et al., 2007 [25]	Aprotinin	30,000 KIU/kg/h/0.7 mL bolus + 10,000 KIU/kg/h/0.2 mL during reperfusion	+15 min (5 min before reperfusion)	20 min/?
Hu et al., 2014 [28]	Remote ischemic preconditioning (RIPC)	4 cycles of 5 min limb ischemia and 5 min reperfusion vs. 4 cycles of limb ischemia and 5 min reperfusion with addition of K_ATP_-blockade	Immediately before the start of the hypovolemia protocol	120 min/72 h
Hu et al., 2016 [27]	Sevoflurane	1.2% vs. 2.2% vs. 3.6%	+60 min	120 min/72 h
Özkan et al., 2008 [30]	Piracetam	800 mg/kg	+60 min	120 min/0 min
Plaschke 2009 [29]	Human mesenchymal stem cells	1 × 106 hMSC	+30 min	30 min/6 days
Wu et al., 2012 [24]	NHE1-Inhibition	3 mg/kg BIIB513 (both for neurological outcome experimental group and organ blood flow experimental group)	Immediately after predefined hypovolemia criteria were met	90 min/72 h

**Table 5:** SYRCLE’s risk of bias tool.

Author/Year	Baseline Characteristics	Allocation Concealment	Random Housing	Blinding (Performance)	Random Outcome Assessment	Blinding (Detection)	Incomplete Outcome Data	Selective Outcome Reporting	Sequence Generation	Other Sources of Bias
Cavus et al., 2008 [16]	+	+	N.A.	+	−	+	+	+	+	+
Cavus et al., 2009 [17]	+	+	N.A.	−	−	+	+	+	+	+
Chien et al., 2011 [18]	+	?	N.A.	−	?	+	+	+	+	?
Eser et al., 2007 [25]	+	−	?	−	−	−	?	?	−	?
Guven et al., 2002 [26]	+	−	N.A.	−	−	−	+	+	−	+
Hu et al., 2014 [28]	+	+	N.A.	+	?	+	+	+	+	+
Hu et al., 2016 [27]	+	−	N.A.	−	−	?	+	+	+	+
Ida et al., 2015 [19]	+	+	N.A.	−	−	+	+	+	+	+
Kishi et al., 2000 [31]	+	−	−	−	−	−	?	?	+	?
Meybohm et al., 2006 [20]	+	?	N.A.	−	?	+	+	+	+	+
Meybohm et al., 2008 [21]	+	−	N.A	−	−	+	+	+	+	+
Meybohm et al., 2007 [22]	+	+	N.A	+	?	+	+	+	+	+
Özkan et al., 2008 [30]	+	−	N.A	−	−	−	+	?	+	+
Plaschke 2009 [29]	+	−	?	?	?	?	−	−	+	−
Vogt et al., 2017 [23]	+	?	N.A	−	?	+	−	+	−	+
Wu et al., 2012 [24]	+	+	?	+	−	+	+	+	+	+

(+) indicates low risk of bias; (−) indicates high risk of bias; (N.A.) Not applicable; (?) indicates unclear risk of bias.

## Appendix A

### Pubmed Search Strategy

1. cerebrovascular circulation [MeSH terms]
2. neuroprotection [tw]
3. neuro* [tw]
4. brain
5. brain protection
6. brain damage
7. cerebral blood flow
8. autoregulation
9. cogni* [tw]
10. 1 OR 2 OR 3 OR 4 OR 5 OR 6 OR 7 OR 8 OR 9
11. shock, hemorrhagic [MeSH terms]
12. hypovol* [tw]
13. hemorrha* [tw]
14. haemorrha* [tw]
15. 11 OR 12 OR 13 OR 14
16. randomized trial
17. randomized trial
18. random*
19. 16 OR 17 OR 18
20. 10 AND 15 AND 19

### CENTRAL Search Strategy

1. MeSH descriptor: [Shock, Hemorrhagic] explode all trees
2. MeSH descriptor: [Hypovolemia] explode all trees
3. hypovol*:ti,ab,kw (Word variations have been searched)
4. hemorrha*:ti,ab,kw (Word variations have been searched)
5. haemorrha*:ti,ab,kw (Word variations have been searched)
6. blood loss:ti,ab,kw (Word variations have been searched)
7. #1 or #2 or #3 or #4 or #5 or #6
8. MeSH descriptor: [Neuroprotective Agents] explode all trees
9. MeSH descriptor: [Cerebrovascular Circulation] explode all trees
10. neuro*:ti,ab,kw (Word variations have been searched)
11. cogniti*:ti,ab,kw (Word variations have been searched)
12. brain damage:ti,ab,kw (Word variations have been searched)
13. #8 or #9 or #10 or #11 or #12
14. #7 and #13

### Web of Science Search Strategy

1. “h*morrhagic shock”
2. “hypovol*mic shock”
3. h*morrhag*
4. hypovol*
5. #1 OR #2 OR #3 OR #4
6. neuroprotect*
7. “cerebr* circulation”
8. “cerebral blood flow”
9. “brain damage”
10. cogniti*
11. #6 OR #7 OR #8 OR #9 OR #10
12. #5 AND #11
13. random*
14. rct
15. trial
16. #13 OR #14 OR #15
17. #12 AND #16

## References

[1] Kauvar D.S., Lefering R., Wade C.E. (2006). Impact of hemorrhage on trauma outcome: An overview of epidemiology, clinical presentations, and therapeutic considerations. J. Trauma.

[2] Peden M., Mcgee K.S., Sharma G. (2002). The Injury Chartbook: A Graphical Overview of the Global Burden of Injuries.

[3] Gutierrez G., Reines H.D., Wulf-Gutierrez M.E. (2004). Clinical review: Hemorrhagic shock. Crit. Care (Lond. Engl.).

[4] Shevell T., Malone F.D. (2003). Management of obstetric hemorrhage. Semin. Perinatol..

[5] Murray C.J., Vos T., Lozano R., Naghavi M., Flaxman A.D., Michaud C., Ezzati M., Shibuya K., Salomon J.A., Abdalla S. (2012). Disability-adjusted life years (dalys) for 291 diseases and injuries in 21 regions, 1990–2010: A systematic analysis for the global burden of disease study 2010. Lancet (Lond. Engl.).

[6] Chouchani E.T., Pell V.R., James A.M., Work L.M., Saeb-Parsy K., Frezza C., Krieg T., Murphy M.P. (2016). A unifying mechanism for mitochondrial superoxide production during ischemia-reperfusion injury. Cell Metab..

[7] Osterbur K., Mann F.A., Kuroki K., DeClue A. (2014). Multiple organ dysfunction syndrome in humans and animals. J. Vet. Intern. Med..

[8] Fantini S., Sassaroli A., Tgavalekos K.T., Kornbluth J. (2016). Cerebral blood flow and autoregulation: Current measurement techniques and prospects for noninvasive optical methods. Neurophotonics.

[9] Rickards C.A., Sprick J.D., Colby H.B., Kay V.L., Tzeng Y.C. (2015). Coupling between arterial pressure, cerebral blood velocity, and cerebral tissue oxygenation with spontaneous and forced oscillations. Physiol. Meas..

[10] Rickards C.A. (2015). Cerebral blood-flow regulation during hemorrhage. Compr. Physiol..

[11] Kudo Y., Ohtaki H., Dohi K., Yin L., Nakamachi T., Endo S., Yofu S., Hiraizumi Y., Miyaoka H., Shioda S. (2006). Neuronal damage in rat brain and spinal cord after cardiac arrest and massive hemorrhagic shock. Crit. Care Med..

[12] Heckbert S.R., Vedder N.B., Hoffman W., Winn R.K., Hudson L.D., Jurkovich G.J., Copass M.K., Harlan J.M., Rice C.L., Maier R.V. (1998). Outcome after hemorrhagic shock in trauma patients. J. Trauma.

[13] Schiffner R., Bischoff S.J., Lehmann T., Rakers F., Rupprecht S., Reiche J., Matziolis G., Schubert H., Schwab M., Huber O. (2017). Redistribution of cerebral blood flow during severe hypovolemia and reperfusion in a sheep model: Critical role of α1-adrenergic signaling. Int. J. Mol. Sci..

[14] Thomas Yeo B.T., Krienen F.M., Sepulcre J., Sabuncu M.R., Lashkari D., Hollinshead M., Roffman J.L., Smoller J.W., Zöllei L., Polimeni J.R. (2011). The organization of the human cerebral cortex estimated by intrinsic functional connectivity. J. Neurophysiol..

[15] Frey C.B., Osborne M.A. (2017). The future of employment: How susceptible are jobs to computerisation?. Technol. Forecast. Soc. Chang..

[16] Cavus E., Meybohm P., Dorges V., Stadlbauer K.H., Wenzel V., Weiss H., Scholz J., Bein B. (2008). Regional and local brain oxygenation during hemorrhagic shock: A prospective experimental study on the effects of small-volume resuscitation with norepinephrine. J. Trauma.

[17] Cavus E., Meybohm P., Doerges V., Hugo H.H., Steinfath M., Nordstroem J., Scholz J., Bein B. (2009). Cerebral effects of three resuscitation protocols in uncontrolled haemorrhagic shock: A randomised controlled experimental study. Resuscitation.

[18] Chien J.C., Jeng M.J., Soong W.J., Hwang B. (2011). Effects of fluid resuscitation on cerebral tissue oxygenation changes in a piglet model of hemorrhagic shock. J. Chin. Med. Assoc. JCMA.

[19] Ida K.K., Otsuki D.A., Sasaki A.T.C., Borges E.S., Castro L.U.C., Sanches T.R., Shimizu M.H.M., Andrade L.C., Auler J.O.C., Dyson A. (2015). Effects of terlipressin as early treatment for protection of brain in a model of haemorrhagic shock. Crit. Care.

[20] Meybohm P., Cavus E., Bein B., Steinfath M., Brand P.A., Scholz J., Dorges V. (2006). Cerebral metabolism assessed with microdialysis in uncontrolled hemorrhagic shock after penetrating liver trauma. Anesth. Analg..

[21] Meybohm P., Cavus E., Dorges V., Weber B., Stadlbauer K.H., Wenzel V., Scholz J., Steffen M., Bein B. (2008). Release of protein s100b in haemorrhagic shock: Effects of small volume resuscitation combined with arginine vasopressin. Resuscitation.

[22] Meybohm P., Cavus E., Bein B., Steinfath M., Weber B., Hamann C., Scholz J., Dorges V. (2007). Small volume resuscitation: A randomized controlled trial with either norepinephrine or vasopressin during severe hemorrhage. J. Trauma.

[23] Vogt N., Herden C., Roeb E., Roderfeld M., Eschbach D., Steinfeldt T., Wulf H., Ruchholtz S., Uhl E., Scholler K. (2017). Cerebral alterations following experimental multiple trauma and hemorrhagic shock. Shock (Augusta Ga.).

[24] Wu D., Russano K., Kouz I., Abraham W.M. (2013). Nhe1 inhibition improves tissue perfusion and resuscitation outcome after severe hemorrhage. J. Surg. Res..

[25] Eser O., Kalkan E., Cosar M., Buyukbas S., Avunduk M.C., Aslan A., Kocabas V. (2007). The effect of aprotinin on brain ischemic-reperfusion injury after hemorrhagic shock in rats: An experimental study. J. Trauma.

[26] Guven H., Amanvermez R., Malazgirt Z., Kaya E., Doganay Z., Celik C., Ozkan K. (2002). Moderate hypothermia prevents brain stem oxidative stress injury after hemorrhagic shock. J. Trauma.

[27] Hu X.W., Wang J.X., Zhang Q.Q., Duan X.W., Chen Z.W., Zhang Y. (2016). Postconditioning with sevoflurane ameliorates spatial learning and memory deficit after hemorrhage shock and resuscitation in rats. J. Surg. Res..

[28] Hu X., Yang Z., Yang M., Qian J., Cahoon J., Xu J., Sun S., Tang W. (2014). Remote ischemic preconditioning mitigates myocardial and neurological dysfunction via k(atp) channel activation in a rat model of hemorrhagic shock. Shock (Augusta Ga.).

[29] Plaschke K. (2009). Human adult mesenchymal stem cells improve rat spatial cognitive function after systemic hemorrhagic shock. Behav. Brain Res..

[30] Ozkan S., Ikizceli I., Sozuer E.M., Avsarogullari L., Ozturk F., Muhtaroglu S., Akdur O., Kucuk C., Durukan P. (2008). The effect of piracetam on brain damage and serum nitric oxide levels in dogs submitted to hemorrhagic shock. Ulus. Travma Acil Cerrahi Derg. Turk. J. Trauma Emerg. Surg. TJTES.

[31] Kishi K., Kawaguchi M., Kurehara K., Inoue S., Sakamoto T., Einaga T., Kitaguchi K., Furuya H. (2000). Hypothermia attenuates the vasodilatory response of pial arterioles to hemorrhagic hypotension in the cat. Anesth. Analg..

[32] Hooijmans C.R., Rovers M.M., de Vries R.B., Leenaars M., Ritskes-Hoitinga M., Langendam M.W. (2014). Syrcle’s risk of bias tool for animal studies. BMC Med. Res. Methodol..

[33] Bulger E.M., Hoyt D.B. (2012). Hypertonic resuscitation after severe injury: Is it of benefit?. Adv. Surg..

[34] Bulger E.M., May S., Kerby J.D., Emerson S., Stiell I.G., Schreiber M.A., Brasel K.J., Tisherman S.A., Coimbra R., Rizoli S. (2011). Out-of-hospital hypertonic resuscitation after traumatic hypovolemic shock: A randomized, placebo controlled trial. Ann. Surg..

[35] Roberts I., Alderson P., Bunn F., Chinnock P., Ker K., Schierhout G. (2004). Colloids versus crystalloids for fluid resuscitation in critically ill patients. Cochrane Database Syst. Rev..

[36] Rossaint R., Bouillon B., Cerny V., Coats T.J., Duranteau J., Fernandez-Mondejar E., Filipescu D., Hunt B.J., Komadina R., Nardi G. (2016). The european guideline on management of major bleeding and coagulopathy following trauma: Fourth edition. Crit. Care (Lond. Engl.).

[37] Bulger E.M., Jurkovich G.J., Nathens A.B., Copass M.K., Hanson S., Cooper C., Liu P.Y., Neff M., Awan A.B., Warner K. (2008). Hypertonic resuscitation of hypovolemic shock after blunt trauma: A randomized controlled trial. Arch. Surg. (Chicago IL. 1960).

[38] Sperry J.L., Minei J.P., Frankel H.L., West M.A., Harbrecht B.G., Moore E.E., Maier R.V., Nirula R. (2008). Early use of vasopressors after injury: Caution before constriction. J. Trauma.

[39] Giraud R., Siegenthaler N., Arroyo D., Bendjelid K. (2014). Impact of epinephrine and norepinephrine on two dynamic indices in a porcine hemorrhagic shock model. J. Trauma Acute Care Surg..

[40] Anand T., Skinner R. (2012). Arginine vasopressin: The future of pressure-support resuscitation in hemorrhagic shock. J. Surg. Res..

[41] Cossu A.P., Mura P., De Giudici L.M., Puddu D., Pasin L., Evangelista M., Xanthos T., Musu M., Finco G. (2014). Vasopressin in hemorrhagic shock: A systematic review and meta-analysis of randomized animal trials. BioMed Res. Int..

[42] Serpa Neto A., Nassar A.P., Cardoso S.O., Manetta J.A., Pereira V.G., Esposito D.C., Damasceno M.C., Russell J.A. (2012). Vasopressin and terlipressin in adult vasodilatory shock: A systematic review and meta-analysis of nine randomized controlled trials. Crit. Care (Lond. Engl.).

[43] Frank F., Broessner G. (2017). Is there still a role for hypothermia in neurocritical care?. Curr. Opin. Crit. Care.

[44] Stocchetti N., Taccone F.S., Citerio G., Pepe P.E., Le Roux P.D., Oddo M., Polderman K.H., Stevens R.D., Barsan W., Maas A.I. (2015). Neuroprotection in acute brain injury: An up-to-date review. Crit. Care (Lond. Engl.).

[45] Lier H., Krep H., Schroeder S., Stuber F. (2008). Preconditions of hemostasis in trauma: A review. The influence of acidosis, hypocalcemia, anemia, and hypothermia on functional hemostasis in trauma. J. Trauma.

[46] Spahn D.R., Bouillon B., Cerny V., Coats T.J., Duranteau J., Fernandez-Mondejar E., Filipescu D., Hunt B.J., Komadina R., Nardi G. (2013). Management of bleeding and coagulopathy following major trauma: An updated european guideline. Crit. Care (Lond. Engl.).

[47] Krueger A., Frink M., Kiessling A., Ruchholtz S., Kuhne C.A. (2013). [emergency room management: In the era of the white paper, s3 guidelines, advanced trauma life support(r) and traumanetwork dgu(r) of the german society of trauma surgery]. Chir. Z. Alle Geb. Oper. Medizen.

[48] Ricci S., Celani M.G., Cantisani T.A., Righetti E. (2012). Piracetam for acute ischaemic stroke. Cochrane Database Syst. Rev..

[49] Verma D.K., Joshi N., Raju K.S., Wahajuddin M., Singh R.K., Singh S. (2015). Metabolic enhancer piracetam attenuates rotenone induced oxidative stress: A study in different rat brain regions. Acta Neurobiol. Exp..

[50] Hwang I.K., Yoo K.Y., An S.J., Li H., Lee C.H., Choi J.H., Lee J.Y., Lee B.H., Kim Y.M., Kwon Y.G. (2008). Late expression of na+/h+ exchanger 1 (nhe1) and neuroprotective effects of nhe inhibitor in the gerbil hippocampal ca1 region induced by transient ischemia. Exp. Neurol..

[51] Cengiz P., Kleman N., Uluc K., Kendigelen P., Hagemann T., Akture E., Messing A., Ferrazzano P., Sun D. (2011). Inhibition of na+/h+ exchanger isoform 1 is neuroprotective in neonatal hypoxic ischemic brain injury. Antioxid. Redox Signal..

[52] Laroni A., de Rosbo N.K., Uccelli A. (2015). Mesenchymal stem cells for the treatment of neurological diseases: Immunoregulation beyond neuroprotection. Immunol. Lett..

[53] Ribeiro T.B., Duarte A.S., Longhini A.L., Pradella F., Farias A.S., Luzo A.C., Oliveira A.L., Olalla Saad S.T. (2015). Neuroprotection and immunomodulation by xenografted human mesenchymal stem cells following spinal cord ventral root avulsion. Sci. Rep..

[54] Iwata Y., Nicole O., Okamura T., Zurakowski D., Jonas R.A. (2010). Aprotinin confers neuroprotection by reducing apoptotic cell death. Asian Cardiovasc. Thorac. Ann..

[55] Iwata Y., Okamura T., Ishibashi N., Zurakowski D., Lidov H.G., Jonas R.A. (2009). Optimal dose of aprotinin for neuroprotection and renal function in a piglet survival model. J. Thorac. Cardiovasc. Surg..

[56] Schifilliti D., Grasso G., Conti A., Fodale V. (2010). Anaesthetic-related neuroprotection intravenous or inhalational agents?. CNS Drugs.

[57] Ishida K., Berger M., Nadler J., Warner D.S. (2014). Anesthetic neuroprotection: Antecedents and an appraisal of preclinical and clinical data quality. Curr. Pharm. Des..

[58] Meller R., Simon R.P. (2015). A critical review of mechanisms regulating remote preconditioning-induced brain protection. J. Appl. Physiol..

[59] Tengfei L., Jiangning W. (2015). Remote ischemic conditioning: A novel way to treat ischemia-related injury of limbs. Med. Hypotheses.

[60] Heusch G. (2015). Molecular basis of cardioprotection: Signal transduction in ischemic pre-, post-, and remote conditioning. Circ. Res..

[61] Pan J., Li X., Peng Y. (2016). Remote ischemic conditioning for acute ischemic stroke: Dawn in the darkness. Rev. Neurosci..

[62] De Vries R.B., Wever K.E., Avey M.T., Stephens M.L., Sena E.S., Leenaars M. (2014). The usefulness of systematic reviews of animal experiments for the design of preclinical and clinical studies. ILAR J..

[63] Russel W., Burch R. (1959). The Principles of Humane Experimental Technique.

[64] Moher D., Shamseer L., Clarke M., Ghersi D., Liberati A., Petticrew M., Shekelle P., Stewart L.A. (2015). Preferred reporting items for systematic review and meta-analysis protocols (prisma-p) 2015 statement. Syst. Rev..

[65] Moher D., Liberati A., Tetzlaff J., Altman D.G. (2010). Preferred reporting items for systematic reviews and meta-analyses: The prisma statement. Int. J. Surg. (Lond. Engl.).

[66] Joanna Briggs Institute JBI Data Extraction form for Experimental/Observational Studies. https://joannabriggs.org/assets/docs/jbc/operations/dataExtractionForms/JBC_Form_DataE_ExpObs.pdf.

[67] Higgins J.P., Altman D.G., Gotzsche P.C., Juni P., Moher D., Oxman A.D., Savovic J., Schulz K.F., Weeks L., Sterne J.A. (2011). The cochrane collaboration’s tool for assessing risk of bias in randomised trials. BMJ (Clin. Res. Ed.).

